# Is climate change a mental health crisis?

**DOI:** 10.1192/bjb.2021.30

**Published:** 2021-08

**Authors:** Daniel Romeu

**Affiliations:** 1 University of Leeds, UK; 2 Leeds and York Partnership NHS Foundation Trust, UK

**Keywords:** Climate change, global warming, mental health, sustainability

## Abstract

The Earth's climate is in a complex state of change as a result of human activity. The interface between climate change and physical health has received significant attention, but its effects on mental health and illness are less understood. This article provides an insight into the psychiatric sequelae of climate change, suggests strategies that psychiatrists can use to take action, and argues that it is their responsibility to do so.

Anthropogenic climate change is irrefutable and has become increasingly difficult to ignore. It is currently considered by experts to represent the largest existential threat to humanity, endangering life itself through environmental degradation, rising ambient temperatures and extreme weather events, among other mechanisms. Efforts to tackle this complex and unpredictable issue have assumed national and international priority, exemplified by the introduction of the 2008 Climate Change Act and the International Paris Agreement, respectively. A global youth movement calling for action on this matter has also received widespread media attention.

The World Health Organization regards climate change as this century's defining issue for health systems. The body of research connecting climate change with health has primarily focused on physical health. Of the literature investigating mental health, the majority either describes effects on vulnerable localities or presents qualitative data,^1^ limiting the universal generalisability of such findings. Individuals with mental disorders are particularly susceptible to the effects of climate change. This is hardly surprising, given its widespread influence on physical, social and economic systems, all of which are key determinants of psychological well-being. It is already apparent that climate change is contributing to an impending and inevitable global mental health crisis.

There is a growing wealth of research examining extreme weather events, including hurricanes, floods and wildfires, in the context of mental health.^1^ Climate experts anticipate an increased periodicity and intensity of such events in a collage of unfolding disasters, rendering these crises harder to predict and respond to. The Australian bushfires and Hurricane Eta are just two examples that caused mass devastation in 2020.

It is helpful to divide the mental health consequences of extreme weather events into direct and indirect.^2^ Such events are associated with mass destruction, and expose the population to direct danger, physical injury and death. Immediate psychological responses include acute stress and grief reactions. The long-term psychopathological patterns that occur following trauma are well understood, and it is recognised that extreme weather events increase the prevalence of post-traumatic stress disorder, depression, anxiety, substance misuse and stress-related relationship difficulties.^3^

We must also consider the destructive impact of extreme weather events on infrastructure, specifically properties, transport services, social support networks and employment. They place additional strain on already overstretched healthcare resources such as community and in-patient mental healthcare systems. In 2005, Hurricane Katrina resulted in a surge in neurotic and affective disorders that disproportionally affected women, young people and those of lower socioeconomic status.^4^ The identification of at-risk groups is essential in ensuring effective prevention and treatment of weather-related mental illness.

Associated with climate change are destructive, enduring and largely irreversible long-term environmental changes – desertification, deglaciation, thawing permafrost, rising sea levels, loss of biodiversity and species extinction, to name a few. Sadness, hopelessness, anxiety and grief reactions are recognised individual psychological responses to environmental degradation.^2^ Societal sequelae include scarcity of resources and involuntary climate-related migration, both of which are likely to result in increased armed conflict and exacerbation of mental health issues. Much of the existing evidence of these responses to climate change come from vulnerable areas, such as coastal and circumpolar regions, which should be regarded as predictions of the future globally.

Global warming is an already demonstrable facet of climate change, and rising temperatures are known to have adverse effects on mental health outcomes. Associations between warmer temperatures and mania in older people, transient affective disorders, substance misuse and psychiatric hospital admissions have all been established empirically.^5^ Furthermore, those affected by mental illness are up to three times more likely to die from a heatwave than those unaffected.^6^ Factors that may explain this vulnerability include poorer baseline physical health, increased isolation and institutionalisation, maladaptive behaviours and the effects of psychotropic medications. Rates of suicide have consistently been found to rise during heatwaves.^1^ Global warming thus represents a key factor influencing psychiatric morbidity and mortality.

Climate change has been shown to increase the risk, frequency and distribution of foodborne, waterborne, vector-borne and zoonotic infectious diseases.^2^ This is likely to be compounded by an uncontrollable surge in global antimicrobial resistance. The profound psychological burden of infectious diseases is plainly exemplified by the COVID-19 pandemic. Restrictive measures, including quarantine, isolation and social distancing, superimposed on vast economic setbacks and a widespread unemployment crisis, have undeniably contributed to a mental health emergency whose full extent is yet to be determined.

Individual psychological responses to COVID-19 are wide-ranging, from anxiety and low mood to insomnia, denial, fear and even anger. The phenomenon of ‘headline stress disorder’, a heightened emotional reaction to seemingly endless news reports that can induce somatic symptoms of anxiety, has been observed throughout the pandemic.^7^ Frontline healthcare workers were particularly susceptible to psychological symptoms during the initial stages of the outbreak,^7^ and this disproportionate impact on our future healthcare workforce will only inflate the magnitude of the crisis. It is probable that ongoing climate change will bring about further pandemics, and COVID-19 provides a means of predicting and mitigating the psychiatric ramifications of new infectious diseases.

The above phenomena are already influencing diagnostic frameworks in psychiatry. There is an advent of integrating new terminology for climate-specific mental disorders into the lexicon, highlighting the growing awareness of climate-driven psychological experiences. ‘Ecoanxiety’ relates to fears about environmental doom and uncertainty, and ‘solastalgia’, a contraction of nostalgia and solace, conceptualises psychological distress secondary to environmental degradation. A range of ‘psychoterratic’ syndromes have since been described, in an attempt to capture the diverse emotional disorders seen in response to climate change.^8^

At present, the DSM-5 and ICD-11 offer no specific references to mental disorders related to climate change, but it is likely that such phenomena will soon be incorporated. Psychiatric phenomenology has historically adapted to contemporary cultural issues, and climate change should be no exception. In 2008, Wolf and Salo described the first patient diagnosed with a putative ‘climate change delusion’.^9^ The patient in question was a 17-year-old boy with a depressive illness, who refused to drink and compulsively checked taps based on the belief that he would deprive others of water. Seasonal affective disorder, now widely regarded as valid and common just 40 years after its first description, is a further example of the evolution of psychiatric frameworks in response to environmental factors.

We have a moral and professional duty as mental healthcare professionals to act on the impending climate-driven health crisis. Every-Palmer et al identify the ‘CARM’ approach – collaborate, advocate, research and mitigate – as a useful tool for conceptualising our opportunities to do so.^3^

Interdisciplinary collaboration with policy makers, healthcare organisations and other stakeholders represents the most comprehensive and effective means of raising awareness of this emerging issue. The *BMJ*, *Lancet* and *PLOS Medicine* have conveyed the responsibility of mental health experts to provide clear information detailing the mental health sequelae of climate change.^3^

It is best practice to treat patients holistically by considering the environmental, social and economic determinants of health and illness. Climate change and its associated complications are a prominent example of such determinants. The literature consistently report a disproportionate impact of climate-related psychological consequences for those with pre-existing mental health issues. We must identify and advocate for vulnerable populations, to ensure equity of resource allocation.

Our understanding of the complex interactions within and between climate change and mental health is in its infancy. Future research in the field should be prioritised to attain a more concrete understanding of these interactions, and to inform the development of effective interventions for both prevention and treatment of climate-related mental health issues. It is crucial that mental healthcare professionals anticipate the inevitable psychological and psychiatric burden of climate change.

The future of global mental health and the planet is in our hands. We should therefore strive to reduce the environmental impact of our institutions and practices. As doctors, our carbon footprint from clinical practice is ten times greater than that from our personal lives.^10^ The Royal College of Psychiatrists’ Sustainability Committee has generated a summary of ten ways to reduce one's footprint in the professional setting^10^ – a good starting point for those willing to make a difference. Mental health professionals take pride in providing individualised care, but we must acknowledge that continuing clinical practice that ignores this issue will contribute to an international mental health crisis. Our decisions will not be forgotten by the patients and psychiatrists of tomorrow.

## About the author

**Daniel Romeu**, MB BChir MA (Cantab) PGCert, is an Academic Clinical Fellow in General Psychiatry at the Leeds Institute of Health Sciences in the School of Medicine, University of Leeds, UK. His clinical work as a Core Psychiatry Trainee is in Acute Inpatient Services at Leeds and York Partnership NHS Foundation Trust, UK.
